# Genetic Variants Affecting FADS2 Enzyme Dynamics and Gene Expression in Cogenetic Oysters with Different PUFA Levels Provide New Tools to Improve Unsaturated Fatty Acids

**DOI:** 10.3390/ijms252413551

**Published:** 2024-12-18

**Authors:** Qingyuan Li, Chaogang Wang, Ao Li, Haigang Qi, Wei Wang, Xinxing Wang, Guofan Zhang, Li Li

**Affiliations:** 1 Shandong Province Key Laboratory of Experimental Marine Biology, Institute of Oceanology, Chinese Academy of Sciences, Qingdao 266071, China; liqingyuan20@mails.ucas.ac.cn (Q.L.); wangcg@qdio.ac.cn (C.W.); ali@qdio.ac.cn (A.L.); qihaigang@qdio.ac.cn (H.Q.); wangxinxing16@126.com (X.W.); 2 Laboratory for Marine Biology and Biotechnology, Qingdao Marine Science and Technology Center, Qingdao 266200, China; gfzhang@qdio.ac.cn; 3 University of Chinese Academy of Sciences, Beijing 101408, China; 4 Key Laboratory of Breeding Biotechnology and Sustainable Aquaculture (CAS), Institute of Oceanology, Chinese Academy of Sciences, Qingdao 266071, China; 5 Laboratory for Marine Fisheries Science and Food Production Processes, Qingdao Marine Science and Technology Center, Qingdao 266071, China; wangwei@qdio.ac.cn; 6 National and Local Joint Engineering Laboratory of Ecological Mariculture, Institute of Oceanology, Chinese Academy of Sciences, Qingdao 266071, China

**Keywords:** LC-PUFA, FADS, genomic variations, gene expression, enzyme dynamics

## Abstract

Long-chain polyunsaturated fatty acids (LC-PUFAs) are crucial for human health and cannot be produced internally. Bivalves, such as oysters, serve as valuable sources of high-quality PUFAs. The enzyme fatty acid desaturase (FADS) plays a key role in the metabolism of LC-PUFAs. In this study, we conducted a thorough genome-wide analysis of the genes belong to the FADS family in *Crassostrea gigas* and *Crassostrea angulata*, with the objective of elucidating the function of the FADS2 and investigating the genetic variations that affect PUFA biosynthesis. We identified six FADS genes distributed across four chromosomes, categorized into three subfamilies. The coding region of FADS2 revealed five non-synonymous mutations that were shown to influence protein structure and stability through molecular dynamics simulations. The promoter region of FADS2 contains ten SNPs and three indels significantly correlated with PUFA content. These genetic variations may explain the differences in PUFA levels observed between the two oyster species and could have potential applications in enhancing PUFA content. This study improves the molecular understanding of PUFA metabolism in oysters and presents a potential strategy for selecting oysters with high PUFA levels.

## 1. Introduction

Polyunsaturated fatty acids (PUFAs) are essential elements of the human diet, playing a crucial role in maintaining cellular integrity and function, as well as regulating metabolic processes and inflammatory responses [1,2]. PUFAs encompass both omega-3 and omega-6 fatty acids, which are important for human health. Fatty acid desaturase (FADS) represents a significant group of fatty acid synthases involved in the desaturation necessary for the biosynthesis of PUFA. This group includes various subfamilies, with FADS2 recognized as the key enzyme in this biosynthetic pathway. The metabolism of PUFAs is complex, involving desaturation and elongation processes, facilitated by a range of enzymes, including FADS2 [3]. *Fads2* encodes the Δ6-desaturase, an enzyme that catalyzes the initial desaturation step in the conversion of short-chain fatty acids into long-chain PUFAs (LC-PUFAs), specifically transforming linoleic acid into gamma-linolenic acid [4]. These desaturation processes are critical for the production of LC-PUFAs. Eicosapentaenoic acid (EPA) and docosahexaenoic acid (DHA) are precursors for biologically active lipid mediators [5]. Given that humans do not synthesize sufficient omega-3 LC-PUFAs, dietary sources are essential, primarily derived from marine fish, which are the important sources of EPA and DHA [2]. Bivalves, recognized for their high-quality lipids, provide an alternative source due to their capacity for endogenous fatty acid synthesis, with their PUFA levels potentially improved through genetic advancements [6,7]. Utilizing molecular breeding to identify individuals with increased desaturase activity offers a promising approach to boost omega-3 fatty acid production. This strategy for producing omega-3 fatty acids from natural sources is characterized by a high level of sustainability and environmental compatibility, along with enhanced economic potential [8].

Enzymes are dynamic systems distinguished by a variety of structures and functions. Understanding the role of conformational dynamics in enzyme evolution is essential not only for deepening our fundamental biochemical insights into enzyme activity but also for practical applications. With the advent of advanced computational tools, biomolecular simulations have become crucial for uncovering enzyme mechanisms and the roots of catalytic activity [9,10]. On one hand, genetic variants can influence the conformational dynamics of enzymes, thereby impacting their catalytic efficiency. On the other hand, variants in the promoter region can alter the binding affinity of transcription factors to downstream genes, affecting transcription rates and potentially leading to reduced gene expression. Ultimately, such changes in gene regulation can significantly influence omega-3 fatty acid levels in organisms. Identifying the genetic variants associated with differences in enzyme dynamics and gene expression among individuals and populations is vital for understanding the varied unsaturated fatty acid profiles and the regulatory mechanisms that could enhance their content through genetic improvement.

Studies in mammals, including humans [11,12,13], goats [14], cows [15], and zebrafish [16], have demonstrated that genetic variants can affect ω-3 fatty acid levels in biological systems by modulating enzyme activity and regulating gene expression. In marine mollusks such as *Octopus vulgaris* [17], *Haliotis discus hannai* [18], *Sinonovacula constricta* [19], *Chlamys nobilis* [20], and *Sepia officinalis* [19], research has mainly concentrated on the specific functions of FADS genes, lacking a comprehensive genome-wide analysis. Additionally, there is currently no established approach in marine biology for identifying individuals with high synthase activity from an enzyme kinetics perspective to enhance PUFA content. While transcript analysis and heterologous expression assays are commonly used to investigate gene function, the effects of genetic variants on enzyme activity and its regulation remain relatively unexamined.

Oysters, as one of the types of marine bivalve shellfish, are an excellent source of PUFAs [21]. They are highly valued as seafood, known for their distinct flavor and texture. Their popularity has increased due to their culinary versatility and nutritional benefits [22,23]. *Crassostrea gigas* and *Crassostrea angulata*, which thrive in different thermal environments, exhibit notable variations in PUFA content and genetic diversity in the *Fads2* gene [24,25]. This divergence provides an opportunity to identify genetic variants responsible for the differences in FADS2 enzyme dynamics and gene expression regulation between the two species. This study conducted a thorough characterization of the Fads gene family in these oyster species through genomic analyses, confirming the functional role of FADS2 in fatty acid desaturation. Genomic variants affecting the stability of the enzyme molecule as well as the activity of the promoter region of the *Fads2* gene have also been identified. These findings will enhance our understanding of the mechanisms governing PUFA synthesis and offer potential genetic tools for selecting individuals with higher PUFA content.

## 2. Result

### 2.1. Sequence and Domain Features of C. Gigas and C. Angulata FADSs

Six putative FADS protein sequences were identified in the genomes of *C. gigas* and *C. angulata*. The lengths of the CgFADS proteins varied from 354 amino acids (Cg25108) to 434 amino acids (Cg15664), with molecular weights ranging from 40.50 kDa (Cg25108) to 51.13 kDa (Cg15664). In contrast, the CaFADS proteins ranged from 353 amino acids (CANg24657) to 444 amino acids (CANg07674), with molecular weights from 40.55 kDa (CANg24657) to 52.32 kDa (CANg07674) (Table 1).

**Figure 1:**
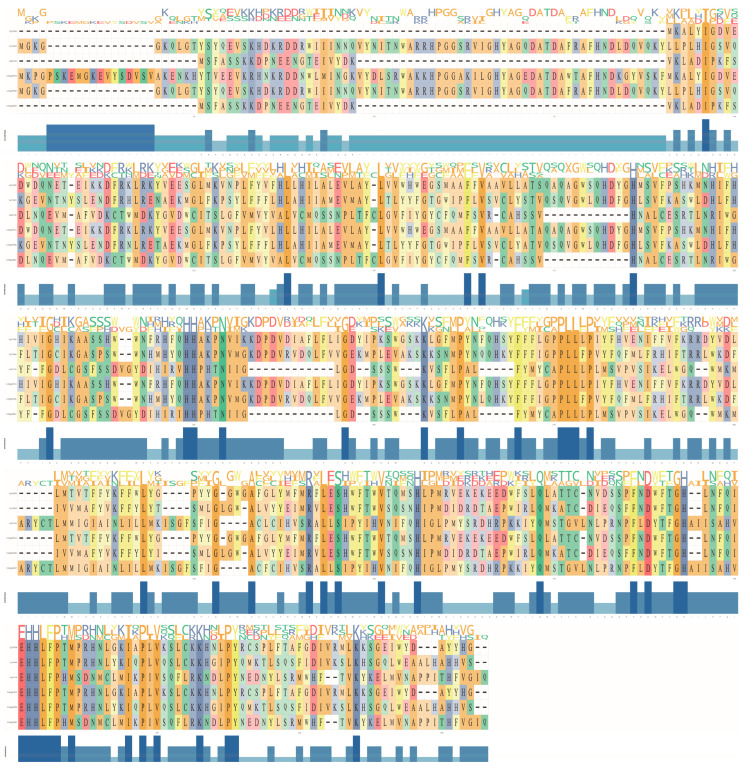
Comparison of amino acid sequences of six FADS2 genes. Through HMM search and local BLAST, six FADS genes were identified. The ‘ggmsa’ package was utilized to create multiple sequence alignments, displaying nucleobase categories and occurrences of each locus above the sequences, while the degree of similarity for each locus was represented in a bar plot.

Multiple sequence alignments revealed moderate overall similarity among the CgFADS proteins, with sequence identity ranging from 0.14 to 0.99. Notably, only three of the fifteen pairwise comparisons among the six proteins had an identity greater than 0.50. The FADS proteins showed a high level of conservation in the ‘EHHLFP’ motif (Figure 1), as well as other identified motifs such as ‘HXXXH’, ‘HXXHH’, and ‘DXXGH’. Predicted subcellular localization indicated that all six proteins were situated in the endoplasmic reticulum (Table 1).

**Figure 2:**
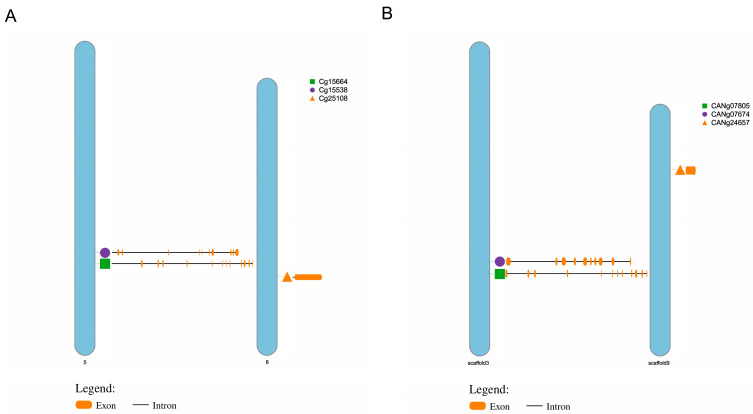
Location on the genome and gene structure of the six FADS genes: (**A**) three FADS genes in *C. gigas*. (**B**) three FADS genes in *C. angulata*.

The three CgFADS genes were distributed across two chromosomes in *C. gigas*: two located on chromosome 5 and one on chromosome 8 (Figure 2A). Similarly, the three CaFADS genes were found on two scaffolds in *C. angulata*, with two on scaffold 3 and one on scaffold 9 (Figure 2B). The gene structures of CgFADS and CaFADS exhibited significant differences: Cg25108/CANg24657 contained a single coding exon, Cg15538/CANg07674 had ten exons, and Cg15664/CANg07805 comprised twelve exons (Figure 2).

### 2.2. Classification of FADS Genes by Phylogenetic Analysis

**Figure 3:**
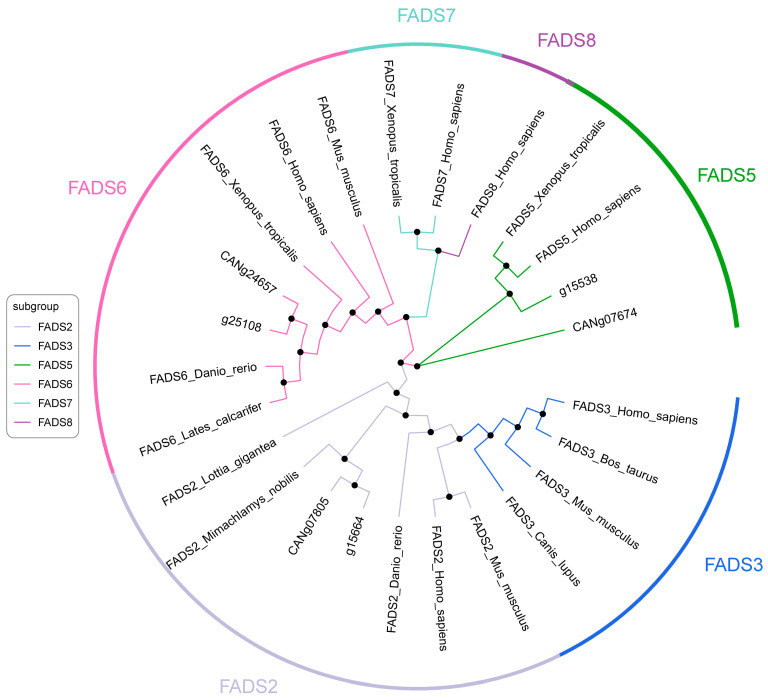
Phylogenetic tree of FADS genes.

Phylogenetic analysis is crucial for determining the subfamily branch to which each gene belongs, facilitating further functional characterization. According to the phylogenetic tree of FADS genes, the six CgFADS and CaFADS genes were categorized into three subfamilies: FADS2, FADS5, and FADS6 (Figure 3). Specifically, Cg15664 and CANg07805 were located within the FADS2 clade, while Cg15538 and CANg07674 clustered together in the FADS5 branch. Finally, Cg25108 and CANg24657 were assigned to the FADS6 subfamily.

### 2.3. Functional Characterization of Oyster FADS2

To thoroughly identify the function of oyster FADS, RNA interference (RNAi) and overexpression studies were performed. A preliminary RNAi experiment targeting oyster FADS2 demonstrated that the siRNA “FADS2-258” significantly reduced CgFADS2 expression after 72 h (Supplementary Figure S1). Consequently, FADS2-258 and a 72 h duration were identified as the most effective siRNA and optimal time point for RNA interference.

**Figure 4:**
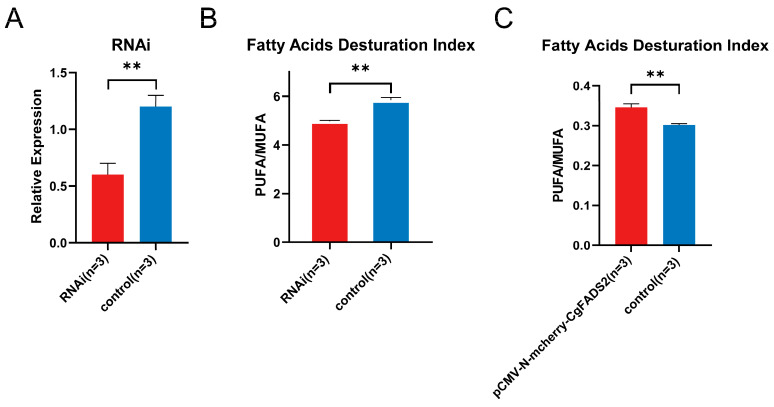
Functional validation of the oyster FADS2 gene. (**A**) Relative gene expression of oyster FADS2 in gill tissues following three repeated injections (72 h) of siRNA-258 during RNA interference experiments (*n* = 3). (**B**) Measurements of fatty acid desaturation indexes during the RNAi experiments (*n* = 3). (**C**) Fatty acid desaturation indexes from the oyster FADS2 overexpression experiment. Significant differences between groups were indicated as follows: ** for *p* < 0.01.

In the formal experiment, CgFADS2 expression was markedly down-regulated following three repeated injections of FADS2-258 compared to the control group (Figure 4A). As a result, the PUFA desaturation indexes also showed a significant decrease (Figure 4B). Additionally, CgFADS2 was cloned and ligated to an overexpression plasmid (pCMV-N-mCherry) and introduced into HK293T cells (Supplementary Figure S2). HEK293T cells that overexpressed CgFADS2 displayed significantly higher PUFA desaturation indexes compared to control cells (Figure 4C). These results highlight the functional role of CgFADS2 in polyunsaturated fatty acid metabolism.

### 2.4. The Substrate Binding Capacity of FADS2

In a previous study, we identified five nonsynonymous genetic variants in CaFADS2 compared to CgFADS2 within the coding region of the FADS2 gene. Predictions of transmembrane structural domains indicated a reduction of one transmembrane domain in CaFADS2 relative to CgFADS2. To further investigate these observations, we analyzed the amino acid polarity and secondary structure of the CgFADS2 and CaFADS2 sequences. Our findings indicated that the genetic variants affected amino acid polarity, potentially influencing the protein’s secondary structure (Supplementary Figure S3).

**Figure 5:**
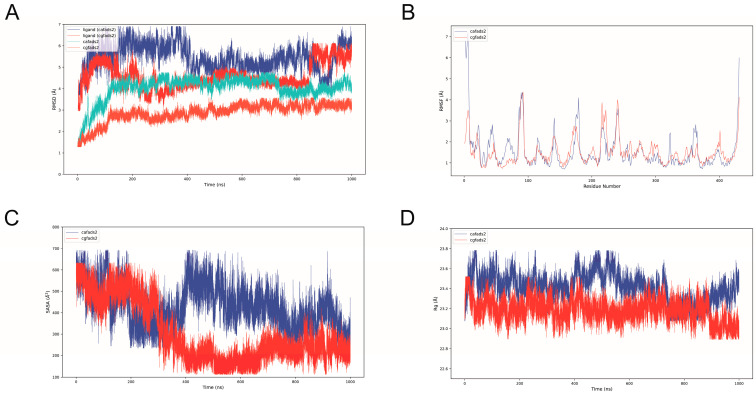
Evaluation of enzyme–substrate binding capacity of FADS2. (**A**) The time course of RMSD values for CgFADS2 (orange), CgFADS2 with substrate (red), CaFADS2 (green), and CaFADS2 with substrate (blue). (**B**) The time course of RMSF values for the two systems. (**C**) The time course of SASA values for the two systems. (**D**) The time course of Rg values for the two systems.

We constructed two systems, CgFADS2 and CaFADS2, to perform 1000 ns molecular dynamics simulations. For assessing the structural stability of these systems, we calculated the root mean square deviation (RMSD) of the Cα atoms of the protein backbone. As depicted in Figure 5A, the RMSD value for the CaFADS2 protein conformation was higher than that of CgFADS2. Notably, even after substrate binding, the RMSD value for CgFADS2 remained lower than that of CaFADS2, indicating that CgFADS2 demonstrates greater stability during substrate binding, which may enhance the synthesis of PUFAs.

The root mean square fluctuation (RMSF) is a key parameter providing insight into how each atom deviates from its equilibrium position over time. Figure 5B shows that the RMSF values for most residues in the CaFADS2 system are elevated to varying extents.

The solvent accessible surface area (SASA) represents the total area of the molecular surface. As illustrated in Figure 5C, the SASA values for the CgFADS2 system are lower than those for the CaFADS2 system. These findings support the subcellular localization of FADS2 in the endoplasmic reticulum membrane, suggesting that CgFADS2 is more stable within the membrane structure.

Finally, the radius of gyration (Rg) evaluates the compactness of the protein structure and quantifies the looseness of the peptide chain during the simulation. Results in Figure 5D indicate that the CgFADS2 protein backbone exhibits greater compactness and rigidity compared to CaFADS2. Collectively, the genetic variants in CaFADS2 may lead to structural differences that affect substrate binding efficiency at the endoplasmic reticulum membrane, thereby influencing its functional properties.

### 2.5. Screening for Potential Causative Genetic Variations

**Figure 6:**
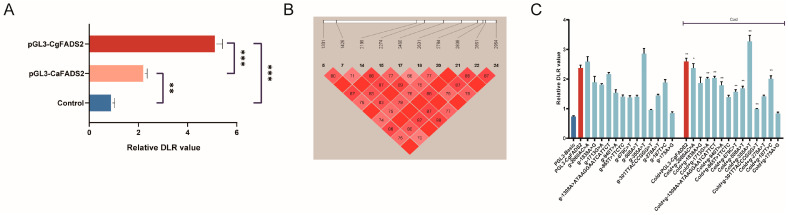
Screening and functional characterization of promoter region variations in FADS2. (**A**) Relative dual-luciferase reporter (DLR) values of cells transfected with the FADS2 gene promoter (*n* = 3). (**B**) Results of linkage disequilibrium block analysis based on genotyping data from *C. gigas* and *C. angulata*. (**C**) Relative DLR values of cells transfected with mutated plasmids in single-site mutation experiments (*n* = 3). The color dark blue indicates the control group, the color red indicates the pGL3-CgFADS2 group, and the color light blue indicates the point mutation group. Significant differences between groups were indicated as follows: * for *p* < 0.05, ** for *p* < 0.01, and *** for *p* < 0.001.

The promoter region is crucial for initiating the transcription process, and mutations in this sequence can significantly affect gene expression. We extracted the promoter regions of FADS2 from *C. gigas* (2215 bp) and *C. angulata* (2197 bp) and cloned them into the pGL3-basic vector. The luciferase reporter result indicated that the FADS2 promoter of *C. gigas* displayed higher transcriptional activity (Figure 6A).

To identify genetic variations, we pooled the amplified promoter sequences to screen for SNPs and InDels within the approximately 2 kb promoter region of oyster FADS2. We identified a total of twenty-two SNPs and four InDels between *C. gigas* and *C. angulata* (*p* < 0.001; Supplementary Table S2). To further validate the significance of these genetic differences, we genotyped additional samples (60 wild *C. gigas* and 60 wild *C. angulata*) using FastNGS sequencing. Our results revealed 13 loci in the FADS2 promoter with significantly different allele frequencies (Table 2).

Moreover, a strong linkage disequilibrium was observed among these 13 variation sites (Figure 6B). These findings underscore the genetic diversity present in the FADS2 promoter region and its potential implications for differential gene expression between the two oyster species.

### 2.6. Functional Analysis of Detected Genetic Variants

To evaluate the effects of the 13 variation sites in the promotor region, we introduced mutations at each site in the pGL3-CgFADS2 and performed a luciferase reporter assay. Eleven genetic variants showed a significant reduction in transcriptional activity compared to the pGL3-CgFADS2 (Figure 6C, Supplementary Figure S4).

Among the genetic variants, seven loci (g.-1713G>A, g.-940T>A, g.-865T>TTCTC, g.-679C>T, g.-508A>T, g.-355A>T, and g.-301TTACCCGGG>T) demonstrated activation in response to cold stimulation. In contrast, two loci (g.-2060AC>A and g.-1308A>ATAAGGAATCATTCT) exhibited inhibitory effects under cold conditions. These results suggest that specific variations in the FADS2 promoter may be important in regulating transcriptional responses to temperature fluctuations, underscoring the functional significance of these genetic variants in the adaptation of oysters to their environments.

## 3. Discussion

FADS family genes play a vital role in PUFA metabolism, as evidenced by studies on gene functions in vertebrates [26]. Marine bivalves, including oysters, scallops, and clams, are particularly recognized for their high PUFA content, mainly consisting of DHA and EPA [27]. Understanding the mechanisms that contribute to this abundant PUFA composition is of great interest in marine biology and aquaculture. With the increasing availability of genomic resources for bivalves, it has become possible to isolate genes involved in PUFA bioprocesses and investigate their functions, as well as the genetic variations that may affect enzyme activity and gene expression regulation. In this study, we scanned the FADS gene family in the whole genomes of *C. gigas* and *C. angulata*. We identified FADS genes related to PUFA metabolism in oysters and examined genetic variations in both coding and promoter sequences that could influence gene expression.

Studies have indicated that directly homologous genes typically share the same function, whereas paralogous genes often fulfill different biological roles [28,29]. A phylogenetic tree analysis demonstrated that the six FADS genes are distributed across three distinct branches, aligning with findings in other species, such as those observed in Haliotis and Sepia [30]. An analysis of gene structure and conserved motifs revealed that all members of the FADS family contain the ‘DXXGH’ motif. Notably, FADS2 and FADS5 displayed identical conserved motifs, ‘HXXXH’ and ‘HXXHH’, respectively, consistent with previous studies [19,31,32]. These three conserved motifs have been recognized as potential enzymatic active sites of FADS, supporting earlier research that emphasizes their functional significance in fatty acid metabolism [33]. Furthermore, FADS6 exhibited significant differences in exon number and motif composition compared to other FADS family members, suggesting it may possess a unique function. These results deepen our understanding of the genetic basis of PUFA metabolism in marine bivalves and highlight the evolutionary adaptations of these species to their environment.

Variations in the coding region of the FADS gene can affect enzyme activity and, in turn, the lipid composition of human tissues. Strong associations have been observed between variants in the human FADS2 gene and levels of PUFAs [34]. Our results indicate that non-synonymous genetic variants can significantly affect the stability of protein–substrate binding. PUFA levels are therefore largely influenced by genetic variation in addition to dietary intake. Additionally, our screening of these variants can help identify individuals with enhanced enzyme activity, potentially leading to increased PUFA production. This research highlights the significance of genetic variation in optimizing enzyme functions, which could result in improved lipid profiles in bivalves. Understanding these genetic influences may offer valuable insights for aquaculture practices aimed at enhancing the nutritional quality of bivalve species.

The FADS2 enzyme is essential for synthesizing PUFAs [35] and has been studied in various mollusk species, including the razor clam [36]. Variants in the promoter region are key components regulating differences in gene expression [37]. However, there have been few studies exploring the role of these elements in mollusks [38,39]. In a previous study, in the non-coding region of environmentally responsive genes, we observed significant sequence differences between *C. gigas* and *C. angulata* [25]. Our analysis uncovered a greater number of mutation sites within the promoter region. Notably, the transcriptional activity of the *C. gigas* promoter was significantly higher than that of the *C. angulata* promoter. A total of 13 genetic variants were identified, showing a strong linkage disequilibrium within the FADS2 promoter, which directly enhanced its transcriptional activity and PUFA content. This indicates that genetic variants in the promoter regulate FADS2 expression to adapt to low temperatures, resulting in higher constitutive expression and PUFA levels in *C. gigas*. Variants in promoter regions are crucial for maintaining constitutive gene expression across different species and populations [40,41,42]. Numerous resequencing studies have identified promoter regions as hotspots for genetic variation [43,44,45,46,47]. In our study, we detected genetic variation in both the coding and promoter regions. The sequence differences in these regions have benefited *C. gigas*, leading to a more stable FADS2 protein structure and increased transcriptional activity. This correlates with the observed phenotypic differences, particularly the higher PUFA content in *C. gigas*.

Although oysters are abundant in PUFAs, the PUFA content can vary significantly among individuals within medium heritability full sibling families [48,49]. Individuals possessing favorable genotypes, characterized by high enzyme activity and gene expression, can be selected for molecular breeding. This study contributes to the field by identifying five coding region variants and thirteen genomic variants in the promoter region that significantly impact FADS2 expression. These variants regulate FADS2 transcription and help explain the differences in PUFA content observed between two species. While further experimental studies are needed, the identified FADS2 gene and promoter variants offer valuable genetic tools for selecting individuals with high enzyme activity, with the goal of producing high-PUFA oysters.

Aquaculture provides a more sustainable method for omega-3 PUFA production compared to wild-caught fishing. Additionally, there is a greater acceptance of food derived from natural sources than that produced through laboratory-based synthetic methods [50]. The genetic enhancement of oysters using genetic engineering techniques represents a more sustainable, environmentally friendly, and economically viable approach for generating omega-3 fatty acids compared to other production methods [51]. This strategy not only supports the health benefits associated with omega-3 PUFA consumption but also fosters a more sustainable aquaculture industry.

## 4. Materials and Methods

### 4.1. Ethics Statement

The oysters utilized in this study were obtained from wild and cultured populations that originated from artificial breeding efforts in Qingdao, China. It is important to note that this study received approval from the Animal Care and Use Committee of the Institute of Oceanology, Chinese Academy of Sciences.

### 4.2. Experimental Animals

A total of 60 *Crassostrea gigas* were collected from Qingdao (35°44′ N) and 60 *Crassostrea angulata* from Xiamen (24°33′ N). Previous research indicated that the average air and sea surface temperature were significantly higher at the southern site compared to the northern site over a two-year span [52]. To evaluate the potential effects of these environmental differences, we conducted a series of common garden experiments [53]. Eggs were harvested from 30 mature females, pooled, and then distributed into 30 beakers for each species. Sperm from 30 mature males was used to fertilize the eggs in each beaker. Four months later, the F_1_ offspring were transferred to the sea off Yantai (37°39′ N, Shandong Province, China). After two months, 15 adult offspring from each species were sampled along with their gill slits for subsequent experiments. Additionally, 30 *C. gigas* were collected for RNA interference experiments. Wild oysters utilized for FastNGS sequencing were gathered from Qingdao (35°44′ N, Shandong Province, China) and Xiamen (24°33′ N, Fujian Province, China) [54].

### 4.3. Identification of Genome-Wide FADS Genes

The FADS domain (Pfam: PF00487) was sourced from the Pfam database and employed as a material for Hidden Markov Model (HMM) search in the genome of *C. gigas* (GCA_011032805.1) and *C. angulata* (GCA_025765675.3) using HMMER software version 3.3.2 [55]. The functional annotation of potential FADS proteins was confirmed by performing a BLASTP search [56] on the NCBI server. Additionally, the integrity of the protein structural domains was assessed using the Conserved Domain Database (CDD) analysis provided by NCBI [57].

### 4.4. Sequence and Structure Features of FADSs

Theoretical isoelectric point and molecular weight predictions for FADS proteins are conducted utilizing the ExPASy server [58]. Multiple sequence comparisons of protein sequence were performed using the R package ‘ggmsa’ (version 1.8.0). [59], followed by calculating pairwise sequence identity. Transmembrane regions of the FADS proteins were predicted using TMHMM [60]. The genomic location and gene structure of each FADS gene were identified through the analysis of *C. gigas* and *C. angulata* genome sequences.

Using the multiple sequence alignments of CgFADS and CaFADS alongside other FADS sequences, a phylogenetic tree was constructed, employing the maximum likelihood method with IQ-TREE and PhyloSuite v1.2.3 software [61,62,63,64,65], incorporating 10,000 bootstrap replicates. The tree was visualized with the R package ‘ggtree’ (version 3.10.1). Subsequently, the FADS genes were classified into subfamilies based on the tree.

### 4.5. RNAi Experiment

The siRNA used in the RNA interference (RNAi) assay was synthesized by GenePharma (Shanghai, China) (sequences are provided in Supplementary Table S1). Thirty individuals of *C. gigas* were acclimatized in a 500 L tank, receiving a daily feed of 4 g/m^3^ of commercial Spirulina powder [24]. After one week, the oysters were anesthetized using a solution of 500 g MgCl_2_ mixed with 5 L of seawater and 5 L of freshwater, then divided into two groups: siRNA group (*n* = 15) and negative control group (*n* = 15) [66].

Next, 100 μL of 10 μg/100 μL siRNA and 10 μg/100 μL negative control strands were injected into the muscles of the respective groups. The optimal duration of interference and the selected siRNA were determined in a preliminary experiment (see Supplementary Figure S2). Based on these results, the most effective siRNA, referred to as “FADS2-258”, was chosen for formal testing, with injections repeated twice after 72 h to promote fatty acid accumulation. After 9 days, gill tissue was collected for gene expression analysis and fatty acid determination.

Quantitative RT-PCR (qRT-PCR) was performed using the ABI 7500 Fast Real-Time PCR System (Applied Biosystems, Waltham, MA, USA) with the Taq Pro Universal SYBR qPCR Master Mix (Vazyme Biotech, Nanjing, China). The primers used for gene expression detection are listed in Supplementary Table S1. The desaturation index of polyunsaturated fatty acids (PUFAs), which reflects the enzyme activity of FADS2 [67], was calculated as follows:

(1) $$PUFA\;desaturation\;index\;=\;\frac{PUFA}{MUFA\;+\;PUFA}$$

### 4.6. Overexpression Experiment

Total RNA was extracted from the gill tissues of *C. gigas* using TRIzol reagent. First-strand cDNA synthesis was carried out with the HiScript^®^ III 1st Strand cDNA Synthesis Kit (Vazyme Biotech, Nanjing, China). The open reading frames (ORFs) of CgFADS2 (100 ng/μL) were amplified using 2×Phanta Max Master Mix (Vazyme Biotech, Nanjing, China) with forward and reverse primers (0.4 μM) that included ApaI restriction sites (New England Biolabs, Ipswich, MA, USA), designed based on the oyster genome (GenBank accession no. GCA_011032805.1) [55]. The amplified DNA fragments were purified from the gel and ligated into a similarly restricted pCMV-N-mCherry vector, resulting in the plasmid construct pCMV-N-mCherry-CgFADS2. The ligation products were transformed into *E. coli* Trelief 5α for recombinant screening via DNA sequencing (Tsingke Biotechnology, Beijing, China).

The pCMV-N-mCherry-CgFADS2 plasmid was purified from *E. coli* using the SPARKeasy Endofree Midi Plasmid Kit (Shandong Sparkjade Biotechnology, Jinan, China). HK293T cells transfected with either pCMV-N-mCherry (control) or pCMV-N-mCherry-CgFADS2 vectors were cultured in high-glucose DMEM medium (2% glucose; Solarbio, Beijing, China) supplemented with 10% fetal bovine serum (Biological Industries, Kibbutz Beit Haemek, Israel) at 37 °C in a 5% CO_2_ incubator. After 2 h of incubation, fluorescence imaging was conducted using a confocal microscope (LSM710, Carl Zeiss, Oberkochen, Germany). After 36 h, HK293T cells were collected, washed, and freeze-dried for subsequent fatty acid analysis. The fatty acid desaturation index was calculated using Equation (1).

### 4.7. Screening and Identification of the Causative Genetic Genetic Variants

We analyzed the sequences within the 2.8 kb FADS2 promoter region from 15 individuals of *C. gigas* and 15 individuals of *C. angulata* using mixed-pool target amplicon sequencing. Primers for amplifying the FADS2 promoter were designed using Primer 5 and synthesized by Tsingke Biotechnology (Supplementary Table S1). PCR amplification was conducted with 2×Phanta Max Master Mix (Vazyme Biotech, Nanjing, China) in a 20 μL reaction volume, which contained 2 μL of DNA template (100 ng/μL), 10 μL of 2×Phanta Max Master Mix, 2 μL of primers (0.4 μM), and 6 μL of H_2_O. The amplification conditions included an initial denaturation at 95 °C for 3 min, followed by 35 cycles of amplification (95 °C for 15 s, 56 °C for 30 s, and 72 °C for 3 min), culminating with a final extension at 72 °C for 5 min. The expected sizes of the DNA products were verified through agarose gel electrophoresis, and the DNA products were purified using the FastPure Gel DNA Extraction Mini Kit (Vazyme Biotech, Nanjing, China).

The concentrations of the purified DNA products were assessed using both NanoDrop 2000 (Thermo Fisher Scientific, Bartlesville, OK, USA) and Qubit 2.0 (Invitrogen, Waltham, MA, USA). Equal volumes of PCR products from *C. gigas* and *C. angulata* were combined into separate pools, each comprising 15 oyster DNA samples. Two pooled DNA samples were created: one for *C. angulata* and one for *C. gigas*. Variant genotypes were identified by Tsingke Biotechnology. The pooled DNA samples were fragmented to approximately 350 bp using a Covaris M220 ultrasonicator (Covaris, Woburn, MA, USA). DNA libraries were prepared utilizing the NEBNext Ultra DNA Library Prep Kit for Illumina (New England Biolabs, Ipswich, MA, USA).

The DNA samples underwent end-polishing, A-tailing, and ligation with full-length adapters for Illumina sequencing, followed by PCR amplification and purification using the AMPure XP system (Beckman Coulter, Brea, CA, USA). The concentration of DNA was measured with a Qubit^®^ 3.0 Fluorometer (Invitrogen, Waltham, MA, USA), and the size distribution was assessed using the Agilent 2100 Bioanalyzer. The libraries were sequenced on an Illumina NovaSeq 6000 platform (Illumina, San Diego, CA, USA), generating paired-end reads. To ensure the quality of the reads, adapter sequences were removed, along with reads containing more than 10% unknown nucleotides (N) or exceeding 40% low-quality bases (Q ≤ 15), using Fastp (version 0.20.1) with default settings [68]. The cleaned reads were then aligned to the reference sequences (GenBase accession no. GB0004783) utilizing the Burrows–Wheeler Aligner [69] with default parameters. SNP calling was conducted using HaplotypeCaller in GATK (version 3.4) [70]. A χ^2^ test was performed based on site sequencing depth to identify significantly different sites between the two groups.

Based on the genotyping results from mixed-pool target amplicon sequencing, variation sites were further confirmed through FastNGS sequencing (Tsingke Biotechnology, Beijing, China). DNA was extracted from 60 wild individuals of *C. gigas* and 60 wild *C. angulata* using the TIANamp Marine Animals DNA Kit (Tiangen Biotech, Beijing, China). Primers were designed with Primer 5 and synthesized by Tsingke Biotechnology (refer to Supplementary Table S1). The PCR procedure followed the same protocol as described previously. After agarose gel electrophoresis and gel extraction, the PCR products were sequenced by Tsingke Biotechnology. The sequences were aligned to the reference sequence (GenBase accession No. GB0004783) and genotyped using VectorNTI (version 8.0) (Invitrogen, Waltham, MA, USA). Linkage disequilibrium analysis (D’ value) was performed using SHEsis [71].

### 4.8. Molecular Dynamics Simulation

Molecular dynamics simulations were carried out using Amber software (Version 22) (San Francisco, CA, USA) [72]. The ff19SB force field was utilized to establish the system’s force field parameters [73]. The TIP3P water model was employed for solvation, and counter ions were added to neutralize the system.

After energy minimization, the system was subjected to a heating phase, increasing from 0 K to 310.15 K (37 °C) over 500 ps. It was subsequently equilibrated in a controlled environment at 310.15 K (37 °C). A 1000 ns molecular dynamics simulation was executed under isothermal and isobaric conditions, while maintaining periodic boundary conditions throughout the simulation. All covalent bonds involving hydrogen atoms were constrained using the SHAKE method. The kinetic data were analyzed using AmberTools23 [74,75].

### 4.9. Luciferase Reporter Assay

The FADS2 promoter regions of *C. gigas* (2215 bp) and *C. angulata* (2197 bp) were amplified through PCR and then cloned into the pGL3-basic vector (MiaoLing Plasmid Platform, Wuhan, China) using the ClonExpress II One Step Cloning Kit (Vazyme Biotech, Nanjing, China). The primers used for amplifying the FADS2 promoter fragments are listed in Supplementary Table S1. The HindIII site was chosen for vector construction (New England Biolabs, Ipswich, MA, USA).

Cell culture was performed as previously described, and transfection was conducted with 400 ng of plasmids containing the FADS2 promoter regions of *C. gigas* and *C. angulata*, along with 100 ng of the pRL-TK Renilla luciferase plasmid (MiaoLing Plasmid Platform, Wuhan, China), using Lipofectamine 3000 (Invitrogen, Waltham, MA, USA). Luciferase activity was evaluated using the Dual-Luciferase Reporter Assay System (Promega, Madison, WI, USA) and measured with a Varioskan Flash multimode reader (Thermo Fisher Scientific, Bartlesville, OK, USA). All experiments included three technical replicates, and firefly luciferase activity was normalized to the Renilla luciferase activity for each sample.

### 4.10. Functional Analysis of Genetic Variants and Measurement of Fatty Acids Content

Each variation site was mutated in the pGL3-CgFADS2 plasmid using the Mut Express^®^ II Fast Mutagenesis Kit V2 (Vazyme Biotech, Nanjing, China). The reaction volume of 50 μL contained 2 μL of pGL3-CgFADS2 as the template, 1 μL of Phanta Max Super-Fidelity DNA Polymerase, 4 μL of primers, 1 μL of dNTP Mix, 25 μL of 2×Max Buffer, and 17 μL of H_2_O. The amplification conditions included an initial denaturation step at 95 °C for 30 s, followed by 35 cycles of amplification (95 °C for 15 s, 60 °C for 15 s, and 72 °C for 8 min), and concluded with a final extension at 72 °C for 5 min.

After verifying the expected size of the PCR products through agarose gel electrophoresis, 1 μL of DpnI was added to the purified PCR products and incubated for 2 h at 37 °C. To restructure the plasmids, a subsequent reaction was conducted, consisting of 4 μL of 5×CE II buffer, 2 μL of Exnase II, 6 μL of the incubation products, and 8 μL of H_2_O, which was incubated for 30 min at 37 °C. The plasmids containing single-site mutations were then transformed into DH5α competent cells (Tsingke Biotechnology, Beijing, China) to confirm the desired mutations through DNA sequencing. The luciferase reporter assay was performed following the same protocol as previously described.

### 4.11. Measurement of Fatty Acids Content

Gas chromatography was employed to quantify the fatty acid content of the cells, adhering to the protocol outlined in our previous study [24]. C19:0 fatty acid methyl ester (Sigma-Aldrich, Burlington, VT, USA) was added as the internal standard, along with a 0.01% butylhydroxytoluene methanol solution to serve as an antioxidant. Total fat was extracted using a dichloromethane–methanol mixture, and the samples were dried under high-purity nitrogen.

Next, 1 mL of a 0.5 M KOH methanol solution was introduced to the mixture and saponified in a water bath at 80 °C for 2 h under nitrogen conditions. After cooling, 1 mL of 14% BF3 methanol solution was added, and the samples were incubated in a water bath at 80 °C for 1 h to facilitate the methyl esterification process. Fatty acid methyl esters were then extracted using n-hexane. The sample volume was adjusted to 0.5 mL and analyzed using an Agilent 7890A gas chromatograph (Agilent Technologies, Santa Clara, CA, USA).

The chromatographic conditions were set as follows: capillary column: DB-FFAP (30 m × 0.32 mm × 0.25 μm); inlet temperature: 220 °C; detector temperature: 280 °C; column temperature program: 150 °C for 1 min, followed by a ramp of 3 °C/min to 220 °C for 33 min.

### 4.12. Statistical Analysis

All statistical analyses were conducted using GraphPad Prism version 8.0.2 for Windows. The normality of distributions was evaluated using the Shapiro–Wilk test, while homogeneity of variance was assessed with Bartlett’s test. Comparisons between two groups were performed using a two-tailed unpaired Student’s *t*-test. For comparisons involving three or more groups, a one-way analysis of variance (ANOVA) was utilized, followed by Tukey’s multiple comparisons test. Notably, relative DLR values during short-term cold stress were analyzed using a two-way ANOVA. Significant differences between groups were indicated as follows: * for *p* < 0.05, ** for *p* < 0.01, and *** for *p* < 0.001.

## 5. Conclusions

In this study, we identified six FADS genes within the genomes of *C. gigas* and *C. angulata*. Phylogenetic analyses showed that these six FADS genes are categorized into three distinct FADS families. We discovered five non-synonymous genetic variants in the coding region of the FADS2 gene between the two species, which may influence the stability of the protein’s binding to its substrate. Additionally, genetic variants in the promoter region, including ten SNPs and three indels, significantly affected the expression levels of FADS2. These variations may contribute to the observed differences in expression regulation patterns and PUFA content between the two species. Overall, this study enhances our understanding of the genetics and mechanisms involved in PUFA metabolism in oysters, providing valuable insights and genetic variations for molecular breeding. This research establishes a foundation for future aquaculture practices aimed at improving the nutritional profile of oysters through genetic selection.

## Figures and Tables

**Table 1:** Sequence characterization and predicted localization of six FADSs.

Gene	Chromosome	Start	End	Aalen ^a^	MolWt ^b^	pI ^c^	GRAVY ^d^	Predicted Location
Cg15538	5	41,053,039	41,058,705	355	42.10	8.66	−0.08	Endoplasmic reticulum
Cg15664	5	43,173,683	43,189,915	434	51.13	9.29	−0.21	Endoplasmic reticulum
Cg25108	8	38,480,631	38,483,654	354	40.50	8.01	0.23	Endoplasmic reticulum
CANg07674	scaffold3	43,527,875	43,533,948	444	52.32	9.10	−0.26	Endoplasmic reticulum
CANg07805	scaffold3	45,988,455	46,000,487	433	51.07	9.29	−0.17	Endoplasmic reticulum
CANg24657	scaffold9	13,001,905	13,002,966	353	40.55	8.01	0.23	Endoplasmic reticulum

^a^ amino acid length. ^b^ molecular weight. ^c^ protein isoelectric point. ^d^ grand average of hydropathy.

**Table 2:** FastNGS sequencing data were used to statistic genotype frequencies.

		*C. gigas*			*C. angulata*			
		0/0	0/1	1/1	0/0	0/1	1/1	*p*-Value
Site 1	g-2060AC>A	56	4	0	10	50	0	<0.0001
Site 2	g-1838A>G	52	7	1	1	36	23	<0.0001
Site 3	g-1713G>A	34	18	8	0	34	26	<0.0001
Site 4	g-1308A>ATAAGGAATCATTCT	2	23	35	1	40	19	0.008
Site 5	g-940T>A	51	5	3	0	4	56	<0.0001
Site 6	g-865T>TTCTC	51	8	1	15	28	17	<0.0001
Site 7	g-679C>T	51	8	1	0	37	23	<0.0001
Site 8	g-508A>T	52	6	2	0	33	27	<0.0001
Site 9	g-355A>T	51	5	4	0	16	44	<0.0001
Site 10	g-301TTACCCGGG>T	53	6	1	0	37	23	<0.0001
Site 11	g-278A>T	52	7	1	0	17	43	<0.0001
Site 12	g-187T>C	35	16	9	2	24	33	<0.0001
Site 13	g-175A>G	52	5	3	2	22	35	<0.0001

## Data Availability

The raw data supporting the conclusions of this article will be made available by the authors on request.

## Supplementary Materials

The following supporting information can be downloaded at: https://www.mdpi.com/article/10.3390/ijms252413551/s1.
