# Supplementary material for: Genetic Variants Affecting FADS2 Enzyme Dynamics and Gene Expression in Cogenetic Oysters with Different PUFA Levels Provide New Tools to Improve Unsaturated Fatty Acids
Source: Int J Mol Sci. 2024 Dec 18;25(24):13551. doi: 10.3390/ijms252413551 (PMC11677070; doi:10.3390/ijms252413551)
Supplement: Supplementary file 1 [file ijms-25-13551-s001.zip › Supplementary material.pdf]

Supplementary Table 1

Primer sequences used in the present study.

Primer name	Usage	Sequences
FADS2-F	ORF cloning	5' - CCGCTCGAGTCTAGAGGGCCCATGGGTAAAGGAGGGAAACAGTT - 3'
FADS2-R	ORF cloning	5' - AGGCTTACCTTCGAAGGGCCCAGAGACATGGTGTGCATGCAGC - 3'
FADS2-258-F	RNAi	5' - GGGAGGUCAAUACAAAUUATT - 3'
FADS2-258-R	RNAi	5' - UAAUUUGUAUUGACCUCCCTT - 3'
mCherry-F	over expression	5' - ctgtacaagggatccaagcttATGGGTAAAGGAGGGAAACAGTT - 3'
mCherry-R	over expression	5' - atcgaattcctgcagaagcttTTAAGAGACATGGTGTGCATGCA - 3'
CgFADS2 promoter-F	upstream promoter element	5' - TGCAACTCTTGGCTAATT - 3'
CgFADS2 promoter-R	upstream promoter element	5' - TGATCCATCGGTCATCCC - 3'
CaFADS2 promoter-F	upstream promoter element	5' - TTTGTTGTTAAGGGTGTGCTACGGA - 3'
CaFADS2 promoter-R	upstream promoter element	5' - CGTGCCCAGTTGGTAATG - 3'

Supplementary Table 2

Statistics of genotype frequency by mixed-pool target amplicon sequencing.

		C.angulata		*C.gigas*		
		REF	ALT	REF	ALT	P value
Site 1	g-2060AC>A	918	2899	2909	0	<0.0001
Site 2	g-1838A>G	1072	6077	2909	0	<0.0001
Site 3	g-1812A>G	1242	5835	2909	0	<0.0001
Site 4	g-1713G>A	1126	5988	2909	0	<0.0001
Site 5	g-1505T>A	1532	5507	6940	0	<0.0001
Site 6	g-1308A>ATAAGGAATCATTCT	2424	2799	3369	0	<0.0001
Site 7	g-1159G>A	1059	4634	4117	0	<0.0001
Site 8	g-1038C>A	841	6195	6915	0	<0.0001
Site 9	g-1031C>A	815	6121	6915	0	<0.0001
Site 10	g-940T>A	1083	5951	7167	0	<0.0001
Site 11	g-865T>TTCTC	2562	3303	6169	0	<0.0001
Site 12	g-843C>T	908	5226	6169	0	<0.0001
Site 13	g-810G>A	747	4813	6169	0	<0.0001
Site 14	g-748C>T	650	3978	6169	0	<0.0001
Site 15	g-679C>T	705	5279	6169	0	<0.0001
Site 16	g-598A>T	817	6513	6169	0	<0.0001
Site 17	g-508A>T	683	6593	6905	0	<0.0001
Site 18	g-378A>G	909	6286	1523	0	<0.0001
Site 19	g-355A>T	873	6293	1523	0	<0.0001
Site 20	g-337G>T	860	6194	1523	0	<0.0001
Site 21	g-301TTACCCGGG>T	1272	5779	1523	0	<0.0001

Site 22	g-278A>T	1235	5882	1523	0	<0.0001
Site 23	g-272C>CTTAA	1267	5806	1523	0	<0.0001
Site 24	g-261T>A	1270	5549	1523	0	<0.0001
Site 25	g-187T>C	1212	5843	1523	0	<0.0001
Site 26	g-175A>G	1211	5748	1523	0	<0.0001
