# Supplementary figures and images for: Genetic Variants Affecting FADS2 Enzyme Dynamics and Gene Expression in Cogenetic Oysters with Different PUFA Levels Provide New Tools to Improve Unsaturated Fatty Acids
Source: Int J Mol Sci. 2024 Dec 18;25(24):13551. doi: 10.3390/ijms252413551 (PMC11677070; doi:10.3390/ijms252413551)

## RNAi

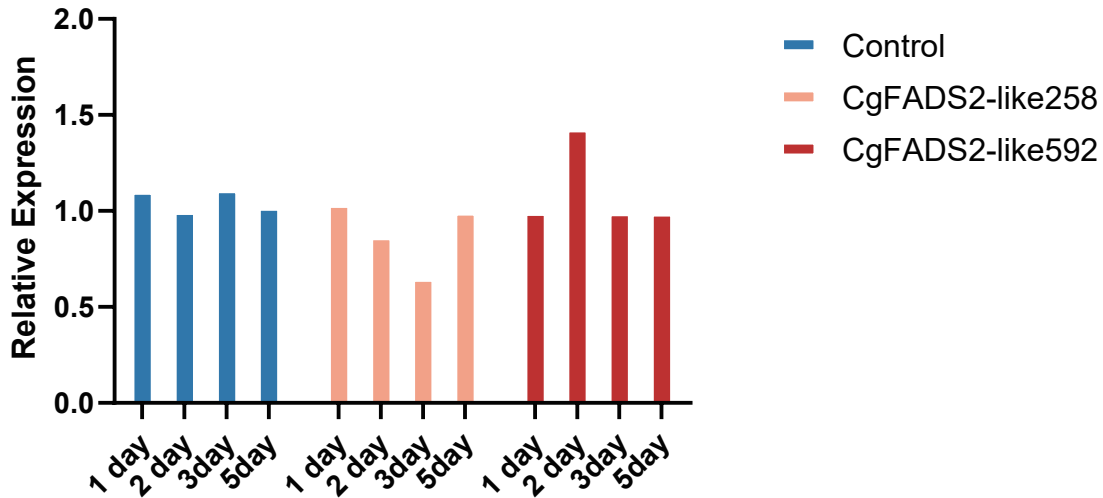

Supplement: Supplementary file 1 [file ijms-25-13551-s001.zip › supplementary figure 1.pdf]

A

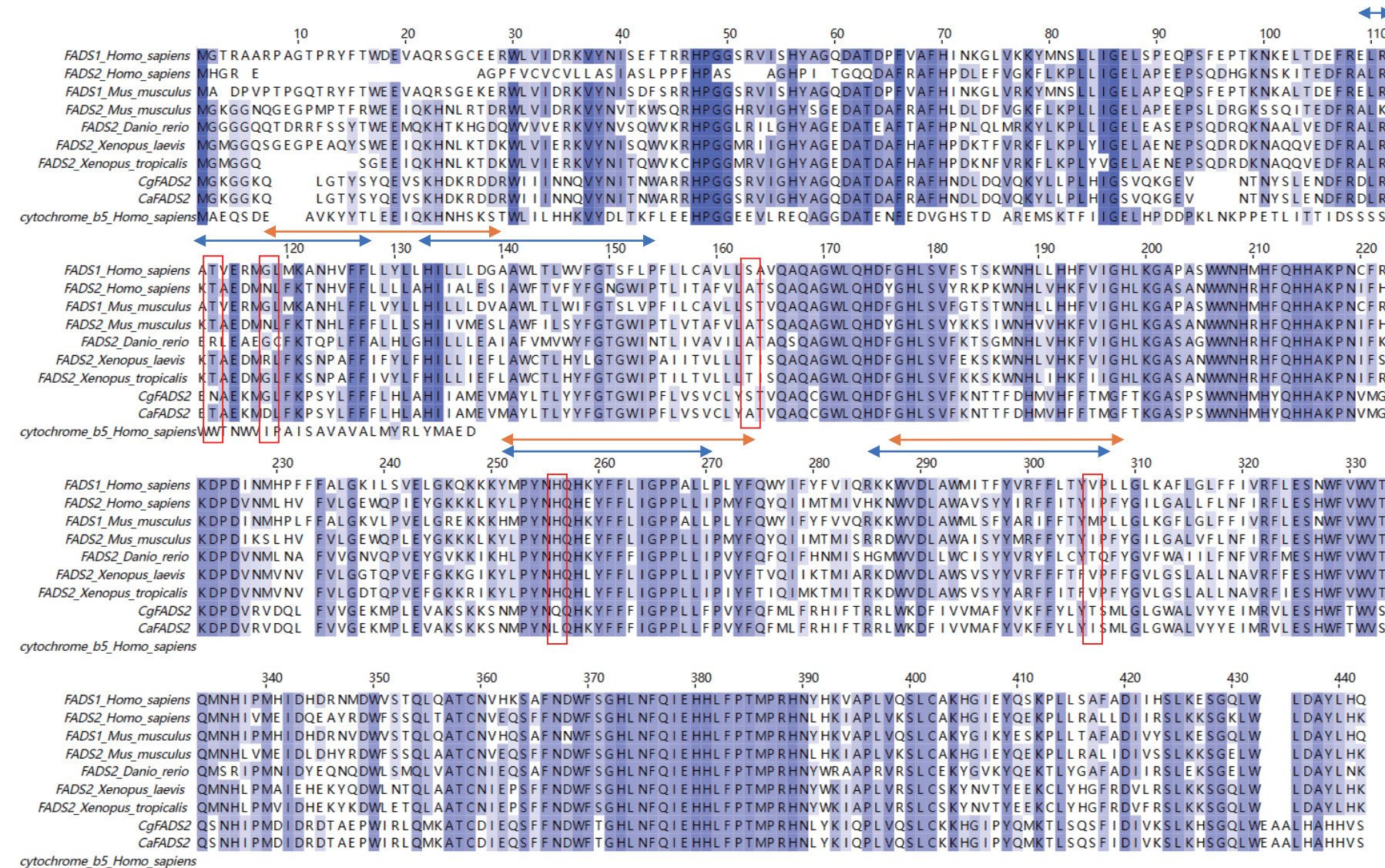

B

## CgFADS2

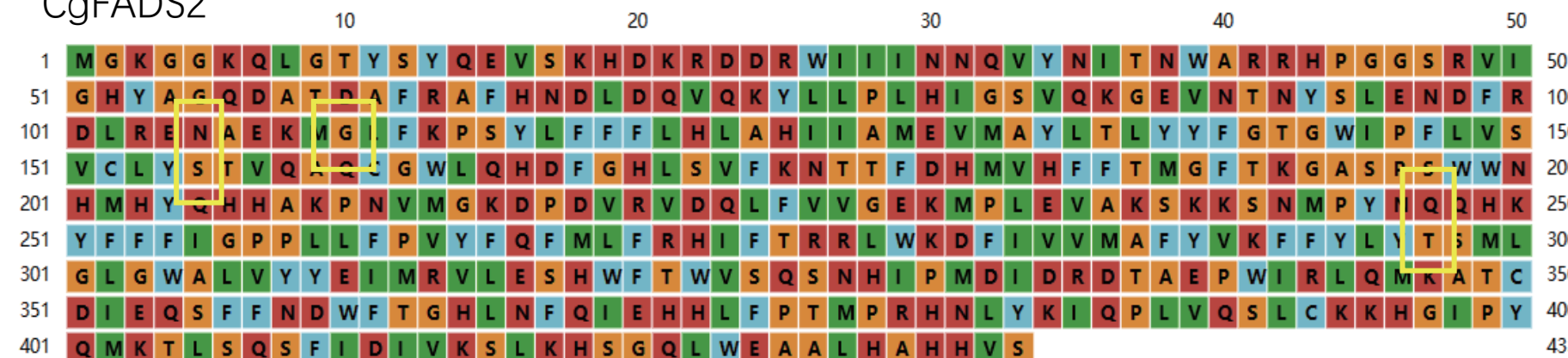

## CaFADS2

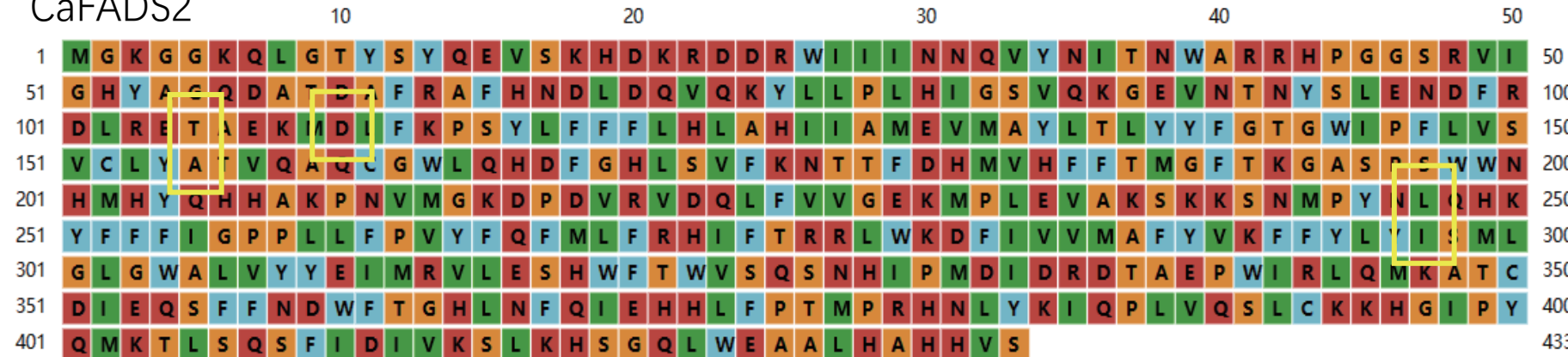

Small nonpolar

Hydrophobic

Polar

Aromatics plus cystiene

C

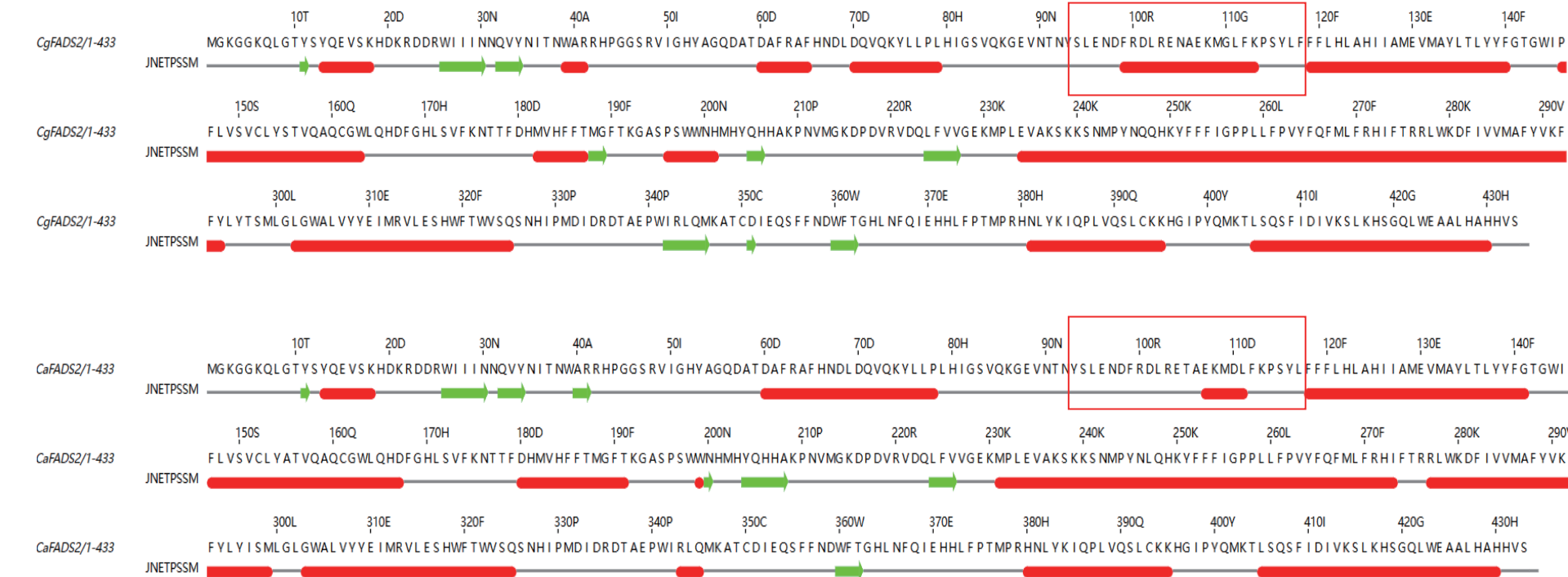

Supplement: Supplementary file 1 [file ijms-25-13551-s001.zip › supplementary figure 2.pdf]

2500

1000

250

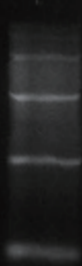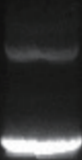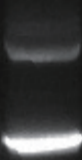

Supplement: Supplementary file 1 [file ijms-25-13551-s001.zip › supplementary figure 3.pdf]

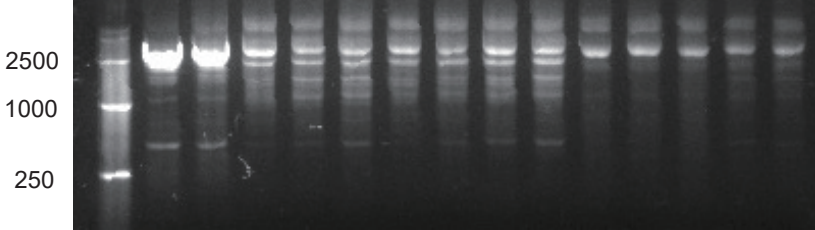

Supplement: Supplementary file 1 [file ijms-25-13551-s001.zip › supplementary figure 4.pdf]
